# Procalcitonin neutralization in sepsis: unresolved effects on mononuclear phagocytes

**DOI:** 10.1515/med-2026-1470

**Published:** 2026-06-30

**Authors:** Franz J. Wiedermann, Wolfgang Lederer, Norbert Mutz, Christian J. Wiedermann

**Affiliations:** Anaesthesiology and Critical Care Medicine, Medical University of Innsbruck, Innsbruck, Austria; InstituInstitute of General Practice and Public Health, Claudiana College of Health Professions, Bolzano, Italy

To the Editor,

Brabenec et al. [1] demonstrate that neutralization of truncated procalcitonin (ProCT) preserves endothelial barrier integrity, reduces vascular dysfunction, and improves organ protection in experimental sepsis. Their study establishes ProCT as a biologically active mediator of endothelial injury rather than a passive biomarker, identifying a Dipeptidyl-Peptidase 4 (DPP4)-dependent activation mechanism and downstream signaling via the calcitonin receptor-like receptor (CRLR)/Receptor Activity-Modifying Protein 1 (RAMP1) complex. ProCT neutralization attenuated IL-17–associated signaling, reduced endothelial transcriptomic activation, preserved nitric oxide bioavailability, and improved vascular and organ function in murine sepsis models.

These findings align with earlier experimental work, summarized by Becker et al. [2], demonstrating improved outcomes following immunoneutralization of ProCT in animal models of systemic inflammation and sepsis. Both studies support ProCT as a therapeutic target within the vascular compartment, with robust transcriptomic, functional, and survival data.

Despite this mechanistic framework for endothelial cells, direct effects of ProCT on human mononuclear phagocytes remain poorly characterized. Mechanistic data on ProCT-driven modulation of monocyte or macrophage function are sparse in contemporary literature. The experimental literature of the past decade provides only limited murine evidence indicating that recombinant ProCT suppresses bone marrow macrophage migration, without receptor identification or defined signaling pathways [3]. No recent studies have systematically examined effects of full-length or truncated ProCT on human monocyte chemotaxis, desensitization, adhesion, or functional deactivation.

This gap warrants attention given that leukocyte migration pathways emerge as prominently regulated in the endothelial transcriptomic profiles reported by Brabenec et al. [1], and that IL-17–dependent signaling mediates ProCT-driven endothelial activation. Endothelial cells regulate leukocyte trafficking through cytokine release, adhesion molecule expression, and barrier remodeling. Whether ProCT directly modulates immune cell migratory behavior alongside its endothelial effects has not been tested.

Our 2002 *in vitro* studies demonstrated concentration-dependent human monocyte chemotaxis at ProCT levels of 1–10 ng/mL [4], matching the plasma concentrations (∼9.5 ng/mL) neutralized by Brabenec et al. in septic mice. Critically, pre-exposure to ProCT abolished subsequent migratory responses – a desensitization pattern characteristic of G-protein-coupled receptor signaling. Whether this desensitization occurs *in vivo* and influences tissue leukocyte infiltration during sepsis has not been investigated. These findings suggested ProCT participation in cross-regulatory mechanisms of leukocyte migration within the inflammatory milieu. This line of investigation has not been pursued in the past two decades, and no modern studies have revisited these functional immune effects using current molecular or cellular approaches.

This evidence gap has direct implications for interpreting the therapeutic effects reported by Brabenec et al. If ProCT induces monocyte desensitization *in vivo* as observed *in vitro*, then antibody neutralization could paradoxically enhance leukocyte tissue infiltration, potentially offsetting vascular benefits in certain clinical contexts. Moreover, the IL-17 reduction following ProCT neutralization may reflect altered monocyte–T-cell crosstalk rather than exclusively endothelial effects. Without cell-specific functional data, it remains unresolved whether immune modulation contributes independently or synergistically to the observed organ protection.

While direct ProCT receptor binding on immune cells remains undemonstrated, related calcitonin-family peptides signal through overlapping CRLR/RAMP complexes and modulate macrophage function [5], 6]. However, antagonism of the CGRP receptor in sepsis worsens outcomes [7], indicating that therapeutic extrapolation from endothelial studies requires cell-type-specific validation.

Current evidence supports a model in which ProCT acts as a vascular toxin that destabilizes endothelial junctions, promotes capillary leakage, and amplifies systemic inflammation [1], 8]. The immune cell component of this model remains incomplete. Without direct functional data on human monocyte responses to ProCT, interpretation of ProCT-targeted interventions is limited to endothelial mechanisms, leaving unresolved whether immune modulation contributes independently or synergistically to observed therapeutic effects.

Mechanistic clarification requires systematic investigation of ProCT signaling in human mononuclear phagocytes, including receptor identification via binding assays, dose–response relationships for migration and cytokine production, and comparative analysis of full-length vs. truncated isoforms. Such studies would resolve whether immune cell effects operate independently or synergistically with vascular protection, directly informing therapeutic strategies targeting ProCT in sepsis.

**Figure 1:**
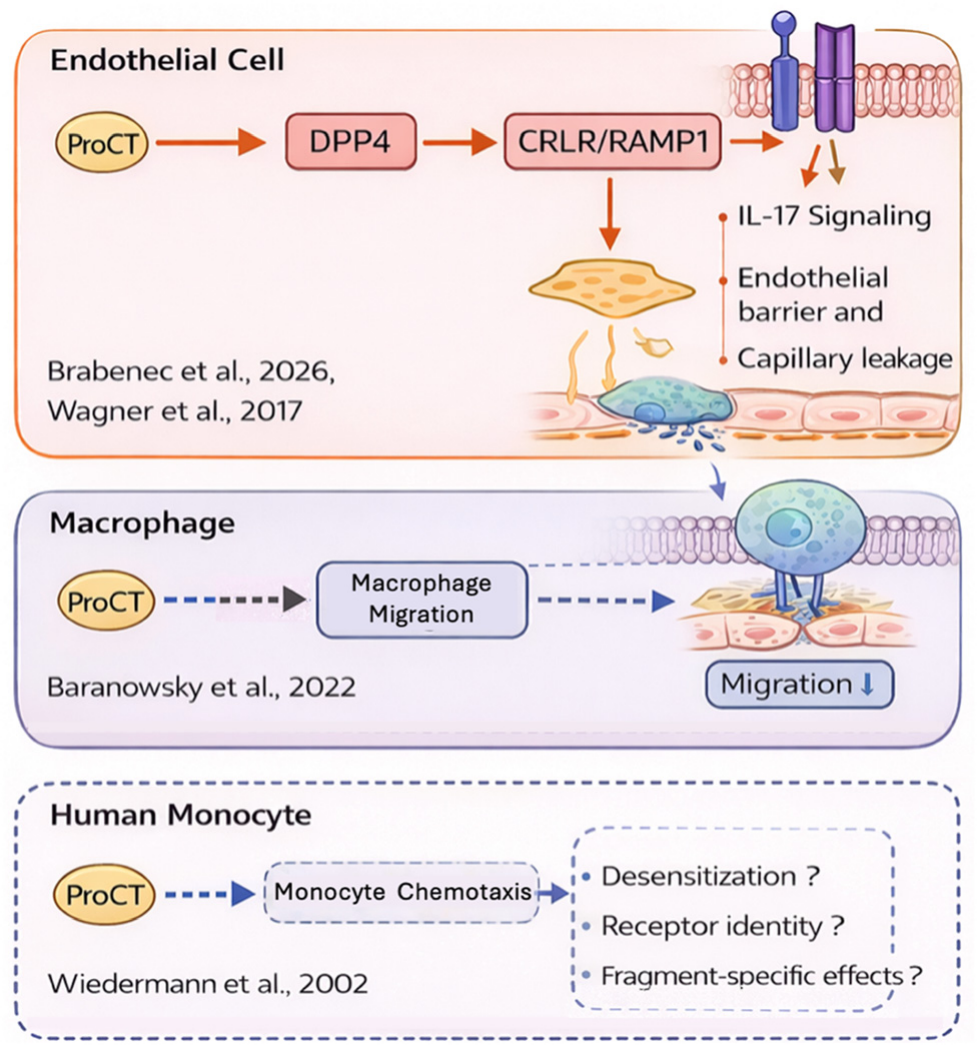
Established and unresolved cellular mechanisms of procalcitonin (ProCT) signaling in sepsis. Solid arrows indicate pathways supported by direct experimental evidence; dashed arrows indicate mechanisms with limited or unresolved evidence. Upper panel: ProCT induces endothelial dysfunction via DPP4-dependent activation and signaling through the calcitonin receptor-like receptor (CRLR)/RAMP1 complex, leading to IL-17–associated signaling, VE-cadherin destabilization, endothelial barrier disruption, and capillary leakage [1], 8]. Middle panel: In murine models, recombinant ProCT (10–1,000 ng/mL) suppresses bone marrow–derived macrophage migration *in vitro* and reduces *in vivo* macrophage recruitment to inflammatory sites; receptor identity and signaling pathways remain undefined [3]. Lower panel: In human monocytes, ProCT induces chemotaxis followed by rapid desensitization; receptor identity, downstream signaling pathways, and fragment-specific effects remain uncharacterized [4].

The work of Brabenec et al. [1] provides compelling evidence for ProCT as a vascular therapeutic target but also highlights an unresolved mechanistic question: whether ProCT modulates innate immune cell function in ways that could augment, oppose, or complicate antibody-based interventions in human sepsis (Figure 1).
